# PNPLA3 rs738409 Polymorphism Predicts Development and Severity of Hepatic Steatosis but Not Metabolic Syndrome in Celiac Disease

**DOI:** 10.3390/nu10091239

**Published:** 2018-09-05

**Authors:** Raffaella Tortora, Antonio Rispo, Anna Alisi, Nicola Imperatore, Annalisa Crudele, Francesca Ferretti, Valerio Nobili, Luca Miele, Nicolò Gerbino, Nicola Caporaso, Filomena Morisco

**Affiliations:** 1 Gastroenterology, Department of Clinical Medicine and Surgery, School of Medicine “Federico II” of Naples, Via S. Pansini 5, 80131 Naples, Italy; raffaellatortora@live.com (R.T.); antoniorispo@email.it (A.R.); nicola.imperatore@alice.it (N.I.); nico46s@libero.it (N.G.); nicola.caporaso@unina.it (N.C.); 2 Research Unit of Molecular Genetics of Complex Phenotypes, Bambino Gesù Children’s Hospital-IRCCS, 00165 Rome, Italy; anna.alisi@opbg.net (A.A.); annalisa.crudele@opbg.net (A.C.); 3 Ferretti: 1. Hepatology, Gastroenterology and Nutrition, “Bambino Gesù” Children’s Hospital, IRCCS, Piazza S. Onofrio 4, 00165 Rome, Italy; Francesca.ferretti@opbg.net (F.F.); valerio.nobili@opbg.net (V.N.); 4 Pediatric Department, University La Sapienza Rome, Piazzale Aldo Moro 5, 00185 Rome, Italy; 5 Department of Internal Medicine and Gastroenterology, Catholic University, 00128 Rome, Italy; luca.miele@unicatt.it

**Keywords:** celiac disease, metabolic syndrome, hepatic steatosis, PNPLA3

## Abstract

Metabolic syndrome (MS) and hepatic steatosis (HS) have been described in patients with celiac disease (CD) after starting a gluten-free diet (GFD), but data on predictive factors for these conditions are scarce. Recently, the patatin-like phospholipase domain-containing protein 3 (PNPLA3) rs738409 has been identified as a key factor for HS development in the general population. The aim of the study was to evaluate the role of PNPLA3 rs738409 in the development of MS and HS in CD patients after starting GFD. Between June 2014 and September 2016, we consecutively enrolled CD patients with HS, while those without steatosis served as a control group. All patients underwent anthropometric and serologic investigations, ultrasonography (US) to assess the degree and severity of HS, and genotyping of the PNPLA3 rs738409 polymorphism. Finally, 370 subjects were enrolled (136 with and 234 without HS). At genotyping assays, the CC genotype was found in 194 subjects (52.4%), the CG genotype in 138 subjects (37.3%), and the GG genotype in 38 subjects (10.2%). At binary logistic regression, only CG and GG alleles were predictive for the development of HS (odds ratio (OR) 1.97; *p* < 0.01 for CG and OR 6.9; *p* < 0.001 for GG). Body mass index (BMI) (OR 3.8; *p* < 0.001) and waist circumference (OR 2.8; *p* = 0.03) at CD diagnosis were the only independent factors for the development of MS. Intergroup comparisons showed that the severe grade of HS was more frequently observed in GG than in CC carriers (74% vs. 11.3%, *p* < 0.001, OR 21.8). PNPLA3 CG and GG carriers with CD have a higher susceptibility to hepatic steatosis, but not to metabolic syndrome. Moreover, patients with GG alleles display more severe forms of HS based on ultrasound.

## 1. Introduction

Celiac disease (CD) is an immune-mediated enteropathy triggered by exposure to gluten in genetically susceptible subjects [1]. Recent evidence has shown that weight modifications are frequent in patients with CD once they start following a gluten-free diet (GFD) [2,3], although data about this topic are still contradictory [4,5,6,7]. These changes are often associated with the development of hypertension, dyslipidemia, diabetes mellitus, hepatic steatosis (HS), and metabolic syndrome (MS) [8,9]. In a previous publication [10], we reported a prevalence of MS in CD patients after one year of GFD of approximately 29%, and of HS in patients with MS of 65%. The increase in weight and the subsequent development of HS and MS in CD could be due to the improvement in intestinal absorption (caused by the exclusion of gluten from the diet) in patients who are in a compensative hyperphagic state [10]. In addition, increased intake of total calories, the macronutrient composition of the diet, may be involved in the pathogenesis of overweight and obesity in patients with CD. Many gluten-free foods are characterized by a glycemic index that is higher than that of equivalent gluten-containing foods [10].

HS has become the most common cause of chronic liver disease, with an estimated prevalence of 20–30% in the general population and 67–75% in obese individuals [11,12]. It is a feature of a wide spectrum of hepatopathies known as nonalcoholic fatty liver disease (NAFLD), ranging from simple steatosis to nonalcoholic steatohepatitis (NASH). In addition, HS can progress to cirrhosis and hepatocellular carcinoma [13]. The pathogenesis of HS is not well known, but it appears multifactorial: in effect, HS has been closely associated with obesity, insulin resistance, diabetes, MS, and dyslipidemia [9,14]. Genetic mutations also play a significant role in predisposition to the development and progression of HS [14,15].

Genome-wide association studies (GWAS) have identified several single-nucleotide polymorphisms (SNPs) associated with increased hepatic fat content [15]. Of these, the patatin-like phospholipase domain-containing protein 3 (PNPLA3) locus on chromosome 22, and especially the nonsynonymous coding SNP rs738409 C/G (I148M), has emerged as a key genetic variant associated with HS [15]. These findings were confirmed by candidate-gene studies assessing the associations with this SNP in several cohorts of different ethnicities [16,17,18,19].

The PNPLA3 gene encodes a transmembrane polypeptide chain containing 481 amino acids. This protein is greatly expressed in hepatocytes, especially on the endoplasmic reticulum and lipid membranes, as well as adipose tissue, and all changes in its expression are narrowly related to nutrient status [20]. Furthermore, metabolic factors such as glucose and insulin regulate the promoter region of the PNPLA3 gene; accordingly, PNPLA3 mRNA levels have been shown to decline after fasting and increase with refeeding in mice. The role of nutrition in the regulation of PNPLA3 has also been highlighted, since the sterol regulatory element binding protein 1c (SREBP-1c), which depends on insulin and glucose, in mouse liver and human hepatocytes is able to regulate the gene [20].

The variant rs738409 is a cytosine-to-guanine substitution that changes codon 148 from isoleucine to methionine. This substitution results in a loss of function of the protein enzymatic activity. This loss of function is a major common genetic determinant of hepatic fat content [21] and progression to chronic liver disease under a large variety of harmful stimuli including severe obesity, visceral adiposity, diet, viruses, iron overload, and excessive alcohol consumption [22].

In detail, Kumari et al. demonstrated that the I148M variant upregulated lipogenic activity, determining an increase in hepatic triglyceride synthesis [23], while Li et al. [24] found that the PNPLA3 I148M variant had several effects on hepatic triglyceride metabolism: increased synthesis of fatty acids and triglyceride, decreased hydrolysis of triglyceride, and reduced triglyceride long-chain polyunsaturated fatty acids. These results suggest that the effects of PNPLA3 I148M variant on hepatic triglyceride levels depend on multiple changes in triglyceride metabolism [20].

However, the role of PNPLA3 I148M variant and the prevalence of PNPLA3 loss of function have never been evaluated in CD, and it is not yet known whether individuals suffering from CD who carry the PNPLA3 I148M variant are more susceptible to chronic metabolic diseases.

The aim of the present study was to evaluate the role of PNPLA3 rs738409 in the development of MS and HS in patients with CD after starting GFD.

## 2. Materials and Methods

### 2.1. Study Population and Study Design

Between June 2014 and September 2016, we consecutively enrolled all adult patients with CD who attended the gastroenterology unit (tertiary center for CD and food intolerance) at Federico II University in Naples, Italy, and who presented hepatic steatosis at ultrasonography (US). During the same period, all consecutive CD patients without steatosis were enrolled as a control group. In accordance with current guidelines [1], CD diagnosis was made in the presence of Marsh ≥ 2 histology associated with both anti-tissue transglutaminase antibody (a-tTG) IgA > 7 U/mL and positive anti-endomysial antibody (EMA) [1]. All enrolled patients were on GFD for at least 1 year.

Exclusion criteria were as follows: history of or ongoing excessive alcohol drinking, defined by an average daily consumption of >20 g for men and >10 g for women; positive tests for hepatitis B surface antigen and antihepatitis C antibody; history of cirrhosis; and clinical, biochemical, or US findings consistent with cirrhosis or other chronic liver diseases. None of the subjects enrolled were on any medication known to promote fatty liver disease.

At diagnosis and follow-up, all patients with CD underwent the following measurements and investigations: weight (kg), height (cm), and waist circumference (cm); blood pressure (mmHg); total cholesterol (mg/dL), low-density lipoprotein (LDL) cholesterol (mg/dL), high-density lipoprotein (HDL) cholesterol (mg/dL), and total triglycerides (mg/dL); blood glucose (mg/dL); blood levels of aspartate (AST, U/L), alanine aminotransferase (ALT, U/L), gamma-glutamyltransferase (GGT, U/L), and ferritin (ng/dL); and abdominal/hepatic ultrasonography (US).

After initial enrollment, all subjects were monitored after 6 months and then every 12 months.

Adherence to GFD was assessed by measuring a-tTG levels and administering a specific questionnaire [25] every 12 months after starting GFD. An expert nutritionist administered the questionnaire and assessed the nutrient intake of all participants.

Blood pressure measurements were carried out in line with European and American guidelines [26,27].

Following the MS criteria of the International Diabetes Federation (IDF) [28], the Adult Treatment Panel III (ATPIII) [29], and European countries [30], diagnosis of MS was made based on the presence of 3 of the following risk factors: central obesity, defined as waist circumference with ethnicity-specific values, specifically for European countries, where waist circumference was ≥94 cm (men) or ≥80 cm (women); blood pressure ≥ 130/85 mmHg (or on antihypertensive treatment); HDL cholesterol < 40 mg/dL (men) or <50 (women) (or on specific treatment); glucose ≥ 100 mg/dL (or previously diagnosed type 2 diabetes); and triglycerides ≥ 150 mg/dL (or on specific treatment). In accordance with the World Health Organization STEPwise approach to Surveillance (WHO STEPS) protocol, waist circumference measurement was made at the approximate midpoint between the lower margin of the last palpable rib and the top of the iliac crest [31]. All serologic tests were carried out in the fasting state, in the morning hours at our laboratories.

Furthermore, the body mass index (BMI) (kg/m^2^) of all patients was recorded at diagnosis and follow-up in line with WHO criteria [32].

Written informed consent was obtained from all participants before the study, which was approved by the local ethical committee (prot. n. 97/2014). The procedures followed were in accordance with the Helsinki Declaration of 1975 as revised in 1983.

### 2.2. Ultrasonography

Liver ultrasonography (US) was performed to assess the degree of steatosis. All US was performed using a 3.5–5 MHz multifrequency probe (Logiq 7 Pro, GE Medical Systems, Milan, Italy) by an expert operator (A.R.) who was blinded to laboratory values. Participants had been fasting for at least 8 h before the procedure. Hepatic steatosis was graded as mild (grade 1), moderate (grade 2), or severe (grade 3).

Mild liver steatosis was defined by a slight increase in liver echogenicity with a slight exaggeration of liver and kidney echo discrepancy. Moderate liver steatosis was characterized by an increase in liver echogenicity and loss of echoes from the wall of the portal vein, with greater posterior beam attenuation and greater discrepancy between hepatic and renal echoes. Severe liver steatosis was characterized by a greater reduction in beam penetration, loss of echoes from most of the portal vein wall, and an even larger discrepancy between hepatic and renal echoes [33,34].

### 2.3. Genetic Analysis

Blood samples were collected from each subject and DNA was extracted by QIAamp Blood MiniKit (Qiagen, Hilden, Germany), according to the manufacturer’s instructions.

The PNPLA3 rs738409 C > G SNP, encoding I148M, was genotyped by using a 5′ nuclease TaqMan assay (Thermo Fisher Applied Biosystems, Foster City, CA, USA). This method combines real-time PCR and mutation detection in a single step.

The probe was cleaved by the 5′ nuclease activity of Taq DNA polymerase only if the specific sequence was successfully amplified. Two TaqMan minor groove binder (MGB) probes were used, one for each allele. One fluorescent dye detector (VIC) was a perfect match for the wild type (allele 1) and the other fluorescent dye detector (FAM) was a perfect match for the mutation (allele 2).

All TaqMan genotyping reactions were carried out in MicroAmp Fast Optical 96-Well Reaction Plate with Barcode (Thermo Fisher Applied Biosystems) in a total volume of 25 µL containing 2× TaqMan Universal PCR Master Mix, No AmpErase UNG (Thermo Fisher Applied Biosystems), 20× SNP Genotyping Assay, and 20 ng DNA.

Reaction plates were cycled on a 7900 HT Fast instrument (Thermo Fisher Applied Biosystems) under the following conditions: 10 min at 95 °C, then 40 cycles of 95 °C for 15 s and 60 °C for 1 min.

Allelic discrimination was performed on the post-PCR product and SDS software was used to analyze the fluorescence. If there was fluorescence from the reporter (VIC) for the wild-type allele, the sample was genotyped as CC. Fluorescence only from the FAM reporter represented homozygosity for the mutant allele and was genotyped as GG. Intermediate fluorescence from both reporters represented the heterozygous population (CG).

Random samples were confirmed by Sanger sequencing (Applied Biosystems 3500 Genetic Analyzer), which provided concordant results in all cases. Positive and negative controls were included on each reaction plate to verify the reproducibility of the results.

### 2.4. Statistical Analysis

Data were analyzed using Statistical Package for the Social Sciences software (v.15.0 SPSS, Inc., Chicago, IL, USA) for Windows and StatsDirect statistical software (v.3.0 StatsDirect, London, UK). We performed a prospective study with a prefixed sample size. We estimated a sample size of 101 cases (hepatic steatosis) and 202 controls (no hepatic steatosis), with a case–control ratio of 1:2, able to offer 80% power (alpha error 5%) to detect a difference of at least 15% in the PNPLA3 prevalence between the 2 groups (when assuming a PNPLA3 prevalence of 20% in control group vs. 35% in case group) [18]. The descriptive statistics used included calculation of mean values and standard deviation (SD) of the continuous variables and the percentages and proportions of the categorical variables. Data were compared using Student’s *t*-test, chi-square test, Fisher’s exact test, and ANOVA, as appropriate. The odds ratio (OR) for quantifying the statistical difference between the dichotomous variables was also calculated. Binary logistic regression was used to examine the relationship between the presence of MS and HS as the dependent variables and the possible predictors as the independent variables. Our regression model used a backward stepwise selection (Wald) method. All the continuous variables were dichotomized as being normal or abnormal (yes/no). The coefficients obtained from the logistic regression analysis were also expressed in terms of odds of event occurrence. A *p* value of less than 0.05 was considered statistically significant.

## 3. Results

### 3.1. Clinical Results

A total of 377 subjects (mean age 36.6 ± 12.5, 74.3% female) who met the inclusion criteria were enrolled in the study. HS was found on ultrasound in 136 patients (36.7%), in accordance with previously reported prevalence of HS in CD [10]; the remaining 234 (63.3%) did not show any sign of liver steatosis. Table 1 summarizes the features of the study population at the time of CD diagnosis according to the subsequent (after a mean follow-up period of 4.8 years) presence or absence of HS.

MS was diagnosed in 66.9% of patients with HS and only 8.9% of patients without liver steatosis (*p* < 0.001). Patients with HS displayed higher rates of hypertransaminasemia, hypertriglyceridemia, hypercholesterolemia, hyperglycemia, diabetes, and hypertension than subjects without steatosis, as well as higher BMI and waist circumference (*p* < 0.001). Table 1 lists the differences between the two groups.

### 3.2. Genetic Results

Our data from the genotyping assay for the analysis of PNPLA3 I148M (rs738409) variants revealed that the CC genotype (wild type) was present in 194 subjects (52.4%), the CG genotype (heterozygous polymorphism) in 138 subjects (37.3%), and the GG genotype (homozygous polymorphism) in 38 subjects (10.2%). No differences were seen among patients with different polymorphisms in terms of anthropometric and serologic variables at the time of CD diagnosis (Table 2). At the end of the follow-up period, patients with CG and GG alleles showed higher rates of hypertension (*p* = 0.001), dyslipidemia (*p* = 0.009), hepatic steatosis (*p* < 0.001), and MS (*p* = 0.001) than patients with the CC genotype (see Table 3).

The intergroup comparison found a statistically significant increase in all metabolic parameters irrespective of PNPLA3 genotype (Table 4).

On the other hand, there were no significant differences in macronutrient intake among subjects with different PNPLA3 genotypes in our cohort, or between patients with or without HS and with or without MS (Table 5).

**Figure 1:**
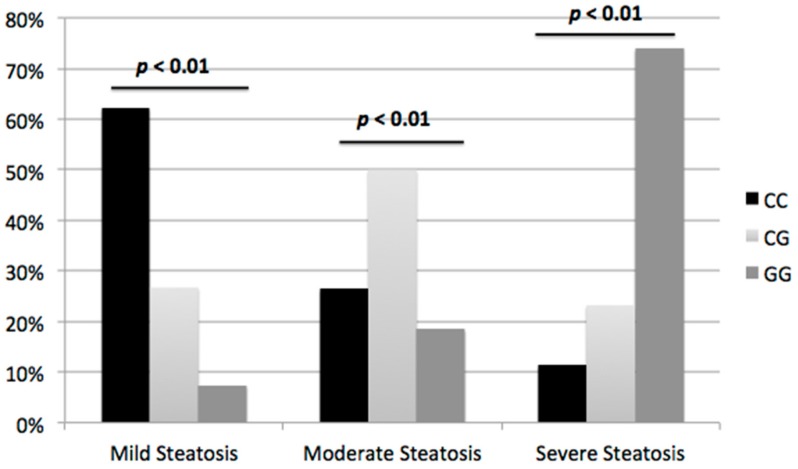
Severity of HS on US in accordance with PNPLA3 genotype. ANOVA test was used to evaluate the differences among groups.

As shown in Figure 1, intergroup comparisons suggest that US images of HS showed higher frequency of severe HS (grade 3) among GG carriers compared to CC carriers: 28 (74%) vs. 22 (11.3%); *p* < 0.001, OR 21.8 (95% confidence interval (CI) 12.3–32.5).

Only 10 patients with severe HS on US and hypertransaminasemia underwent liver biopsy; histologic examination revealed a diagnosis of NASH in all of them. Of those 10 patients, eight (80%) were GG carriers, while the remaining two had CG alleles.

### 3.3. Predictive Factors for Metabolic Syndrome and Hepatic Steatosis in Celiac Population

The results of the logistic regression performed to establish the effect of gender, BMI, G allele, GG alleles, waist circumference, hypertension, hyperglycemia, hypertransaminasemia, hypercholesterolemia, and hypertriglyceridemia on the likelihood of developing HS or MS were as follows: only G and GG alleles were predictive for the development of HS (OR 1.97; 95% CI 1.4–2.7; *p* < 0.01 for G carriers and OR 6.9; 95% CI 4.2–9.5; *p* < 0.001 for GG carriers), while higher BMI (OR 3.8; 95% CI 1.6–5.2; *p* < 0.001) and higher waist circumference (OR 2.8; 95% CI 1.9–5.4; *p* = 0.03) at CD diagnosis were the only independent factors for the development of MS (Table 6).

No statistically significant differences were observed in terms of compliance with diet, as assessed by a questionnaire.

## 4. Discussion

NAFLD has become the most common cause of chronic liver disease, with an estimated prevalence of 20–30% in the general population and 67–75% in obese individuals. Previous findings reported by our research team [10] showed a high prevalence of nonalcoholic HS and MS in CD patients following one year of GFD. However, data about predictive factors for HS and MS other than GFD in this population are unknown.

In 2008, Romeo and colleagues [16] first reported a genome-wide association study (GWAS) to explore the genes associated with susceptibility to HS. They showed that the PNPLA3 I148M genetic variant was strongly associated with increased liver fat content, and that the association remained highly significant after adjusting for BMI, diabetes, ethanol intake, and global and local ancestry. In addition, the PNPLA3 I148M variant was also found to be associated with elevated serum aminotransferase levels [16], increased computed tomography–measured hepatic steatosis, and histologic HS [35].

In vitro studies have shown that the I148M allele encodes for a loss-of-function variant that predisposes to steatosis by reducing triglyceride hydrolysis in hepatocytes and modulating adipocyte size [36]. Recent studies have hypothesized a direct role for PNPLA3 in the activation of hepatic stellate cells, and this mechanism could be the basis for steatosis and fibrogenesis; however, further studies are needed in order to demonstrate this relationship [37].

The present study, aiming to evaluate the role of PNPLA3 rs738409 variant in the development of HS and MS in a large series of patients with CD after starting GFD, demonstrated that G and GG alleles were independent predictive factors for HS development (OR 1.97; *p* < 0.01 for G carriers and OR 6.9; *p* < 0.001 for GG carriers). BMI (OR 3.8; *p* < 0.001) and waist circumference (OR 2.8; *p* = 0.03) at CD diagnosis were the only independent factors associated with the development of MS. It follows that our study confirms the results from previous studies, which showed that the presence of the G allele in PNPLA3 rs738409 increased the risk of HS [16,17,18,19].

In a large-scale Chinese community-based population study [38], the GG genotype in PNPLA3 rs738409 was associated with a nearly 1.5-fold increase in the prevalence of HS compared to the CC genotype. A recent meta-analysis by Sookoian et al. [39] established that in nonceliac populations, the GG genotype was associated with a nearly 3.3-fold increase in the prevalence of HS compared to the CC genotype. Our study does not demonstrate any correlation between the G allele and prevalence of MS, although a role for the G allele in MS has been hypothesized [39,40]. In our study, the prevalence of HS, stratified by BMI, in subjects with different PNPLA3 genotypes showed no statistically significant difference between groups. Notably, HS associated with the PNPLA3 G allele is not necessarily characterized by features of MS [38], which is ultimately distinct from obesity-related HS. In this setting, HS is considered as the expression of MS in the liver [9].

Although a few studies have hypothesized an association between the PNPLA3 I148M variant and MS, for example mediated by insulin resistance [41], other studies have failed to confirm this [39,42]. Mangge et al. [43] concluded that the PNPLA3 rs738409 polymorphism was associated with increased ALT in young people, and that it was more frequent in people of all ages with obesity and MS. By contrast, one recent study [44] found that the PNPLA3 rs738409 genotype was the strongest predictor of a diagnosis of HS in subjects who did not have MS. Romeo et al. [16] found no association between the PNPLA3 I148M allele and BMI or indices of insulin sensitivity. In addition, Del Ben et al. [45] showed that the PNPLA3 I148M gene variant was associated with a lower prevalence of MS and reduced cardiometabolic risk in a large number of individuals with HS. In a recent meta-analysis [39], all included studies showed no significant difference among PNPLA genotypes in terms of BMI, glucose and insulin levels, and homeostasis model assessment of insulin resistance.

In our study, US images showed that severe HS (grade 3) was more frequently observed in GG than in CC carriers (74% vs. 11.3%, *p* < 0.001, OR 21.8). This result can be compared with findings from other studies that demonstrated an association between PNPLA3 polymorphism and histologic severity of HS.

Rotman et al. [46] confirmed the association of the PNPLA3 rs738409 G allele in a large cohort of patients with histologically proven HS. Another large study showed that PNPLA3 variation conferred a markedly increased risk of severe histologic features of HS, without any strong effect on elements of MS [41]. Finally, a meta-analysis of 16 studies across different populations showed a strong influence of the PNPLA3 I148M variation not only on liver fat accumulation, but also on susceptibility to more aggressive disease, with GG carriers having a greater risk of experiencing more extensive necro-inflammation and developing fibrosis, compared to PNPLA3 I148I homozygotes [39].

Several studies demonstrated that GFD, although an indispensable treatment for CD, has potentially negative effects on nutritional status [47]. Wild et al. [48] reported that British female CD patients consumed more energy from all macronutrients when compared with a nonceliac population, attributing this to higher intake of sweet snacks that were richer in saturated fat. There is clinical evidence indicating a high intake of simple sugars, proteins, and saturated fat and intake of complex carbohydrates and fiber in such diets [10]. In addition, increased intake of total calories, the macronutrient composition of the diet, may be involved in the pathogenesis of overweight and obesity in patients with CD. Therefore, GFD might influence data interpretation. Moreover, a recent study by Lebwohl et al. [49] concluded that avoiding gluten might result in reduced consumption of beneficial whole grains, which may affect cardiovascular risk. Promoting GFD among people without CD should not be encouraged. In the present study, we were able to show that there were no differences in terms of macronutrient intake in CD patients with different PNPLA3 genotypes with or without steatosis, avoiding the bias of diet and underlining the key role of PNPLA3.

Our work has some limitations that we now briefly discuss. First, the diagnosis of HS was primarily based on ultrasonographic findings; this limitation is unavoidable, as diagnosis based on a more invasive technique such as liver biopsy would be ethically questionable. However, a recent meta-analysis [50] showed that US is an accurate, reliable imaging technique for the detection of fatty liver. In our sample, only 10 patients with severe HS on US and hypertransaminasemia underwent liver biopsy, which confirmed the diagnosis of NASH in all of them. Second, the lack of a “healthy subjects” control group in our study may represent a procedural limitation. However, the availability of several published studies carried out with populations of the same ethnicity as ours allows for useful comparisons [14,18,19,20,36]. We decided to exclude any patients with advanced liver disease in order to avoid heterogeneity within our population. In fact, the presence of cirrhosis could have altered the CD-related metabolic parameters (i.e., transaminases, cholesterol, ferritin, also BMI in case of decompensated cirrhosis/ascites). However, the role of PNPLA3 in predicting the risk of cirrhosis has been already described [51].

The assessment of compliance with diet and caloric intake was made on the basis of CD serology, compliance questionnaires [25], and food questionnaires with a patient-tailored approach on the basis of daily caloric needs. Although establishing compliance with diet and caloric intake for CD patients is always challenging for physicians, we decided to use these simple and validated approaches [52], even though they are susceptible to bias. Finally, it has been recently demonstrated [53] that adherence to GDF was higher when foods were prescribed than when patients did not receive foods by prescription, thus highlighting the remarkable impact on the quality of GFD of having a nutritionist involved.

## 5. Conclusions

In conclusion, our results show that PNPLA3 G and GG carriers with CD have higher susceptibility to HS but not to MS. Furthermore, patients with GG alleles display more severe disease in terms of HS grade based on US imaging. These findings highlight that analysis of the PNPLA3 I148M polymorphism may be a valuable tool in the overall management of patients suffering from CD.

## Figures and Tables

**Table 1:** Features of the study population at the time of celiac disease diagnosis and after follow-up period according to presence/absence of hepatic steatosis (HS).

	At Diagnosis				At Follow-up			
	All (370)	Steatosis (136)	No Steatosis (234)	*p*	All (370)	Steatosis (136)	No Steatosis (234)	*p*
Male/Female, *n* (%)	95 (25.7)/275 (74.3)	43 (31.7)/93 (68.3)	52 (22.3)/182 (77.7)	0.04	95 (25.7)/275 (74.3)	43 (31.7)/93 (68.3)	52 (22.3)/182 (77.7)	0.04
Age, years (mean ± SD)	31.3 ± 14.4	32.1 ± 14.7	31.1 ± 15	0.2	36.6 ± 12.5	36.9 ± 11.9	36.3 ± 12.1	0.4
Follow-up period, years (mean ± SD)	-	-	-	-	4.7 ± 1.2	4.8 ± 1.2	4.7 ± 1.3	0.8
a-tTG IgA, U/mL (mean ± SD)	112.6 ± 89.3	97.8 ± 72.5	102.1 ± 88.6	0.7	5 ± 1.3	6 ± 1.2	4 ± 1.4	0.2
EMA positivity, *n* (%)	370 (100)	136 (100)	234 (100)	1	0 (0)	0 (0)	0 (0)	1
AST, U/L (mean ± SD)	24.1 ± 23.8	26.7 ± 31.1	22.7 ± 18.2	0.1	19.2 ± 6.4	22.3 ± 7	17.9 ± 6.2	<0.001
ALT, U/L (mean ± SD)	25.6 ± 23.7	28.4 ± 22.3	24.1 ± 24.4	0.09	22.2 ± 8.3	24.8 ± 11.5	18.1 ± 8.6	0.001
Hypertransaminasemia, *n* (%)	39 (10.5)	20 (14.7)	19 (8.1)	0.04	20 (5.4)	14 (10.2)	6 (2.5)	0.002
GGT, U/L (mean ± SD)	16.5 ± 10.4	18.5 ± 11.3	15.4 ± 9.7	0.007	16.8 ± 9.8	19.2 ± 11.3	15.4 ± 7.4	<0.001
Triglycerides, mg/dL (mean ± SD)	90.1 ± 37.8	97.5 ± 37	85.8 ± 37.7	0.004	121 ± 50.3	138.4 ± 52.7	101.2 ± 48.5	<0.001
Hypertriglyceridemia, *n* (%)	17 (4.3)	9 (6.6)	8 (3.4)	0.1	72 (19.4)	50 (36.7)	22 (9.4)	<0.001
Total cholesterol, mg/dL (mean ± SD)	163.7 ± 35.2	168.5 ± 35.8	160.9 ± 34.6	0.04	179.4 ± 29.3	192.6 ± 28.4	170.5 ± 30.4	<0.001
Hypercholesterolemia, *n* (%)	46 (12.4)	25 (18.3)	21 (8.9)	0.008	92 (24.8)	61 (44.8)	31 (13.2)	<0.001
HDL, mg/dL (mean ± SD)	54.4 ± 13	55.1 ± 12	53.9 ± 13.6	0.3	54.2 ± 12.8	51.3 ± 14.9	56.2 ± 11.6	0.001
LDL, mg/dL (mean ± SD)	117.2 ± 39.1	135.7 ± 52.6	109.3 ± 28.9	0.01	119.8 ± 38.1	124.8 ± 40.2	116.2 ± 34.8	0.4
Fasting glucose, mg/dL (mean ± SD)	84.1 ± 19.8	85.8 ± 23.6	83.1 ± 17.2	0.2	88.7 ± 13.4	92.5 ± 16.2	83.7 ± 11.1	<0.001
Hyperglycemia, *n* (%)	8 (2.2)	5 (3.6)	3 (1.2)	0.1	30 (8.1)	20 (14.7)	10 (4.2)	<0.001
Ferritin, ng/dL (mean ± SD)	28.7 ± 38.7	28.9 ± 34.5	28.5 ± 41	0.9	48.8 ±42.5	54.7 ± 46.1	43.1 ± 39.5	0.014
Weight, kg (mean ± SD)	64.1 ± 13.7	66.4 ± 15.4	62.7 ± 12.4	0.01	68.7 ± 13.9	71.9 ± 15	65.8 ± 11.2	<0.001
Height, cm (mean ± SD)	165.4 ± 10.2	165.4 ± 8.3	165.3 ± 11.2	0.9	165.4 ± 10.2	165.4 ± 8.3	165.3 ± 11.2	0.9
BMI, Kg/m^2^ (mean ± SD)	23.2 ± 3.6	24.1 ± 4.3	22.7 ± 3.1	0.001	24.8 ± 3.5	25.9 ± 4.2	23.7 ± 3	<0.001
Waist circumference, cm (mean ± SD)	85.1±10.9	88.2 ± 12.3	83.1 ± 9.3	0.001	89.2 ± 10.4	92.4 ± 12.9	85.8 ± 9.4	<0.001
Hypertension, *n* (%)	38 (10.3)	15 (11)	23 (9.8)	0.5	113 (30.5)	87 (63.9)	26 (11.1)	<0.001
Dyslipidemia, *n* (%)	40 (10.8)	18 (13.2)	22 (9.4)	0.2	118 (31.9)	89 (65.4)	29 (12.3)	<0.001
Diabetes, *n* (%)	2 (0.5)	1 (0.7)	1 (0.4)	0.4	17 (4.6)	13 (9.5)	4 (1.7)	0.001
Metabolic syndrome, *n* (%)	28 (7.2)	13 (9.5)	14 (5.9)	0.08	112 (30.2)	91 (66.9)	21 (8.9)	<0.001

Statistical analysis between steatosis and no steatosis was performed by using Student’s *t*-test, chi-square test, Fisher’s exact test, when indicated. *n*, number; SD, standard deviation; a-tTg IgA, anti-tissue transglutaminase antibody; EMA, anti-endomysial antibody; AST, aspartate aminotransferase; ALT, alanine aminotransferase; GGT, gamma-glutamyl transpeptidase; HDL, high-density lipoprotein; LDL, low-density lipoprotein; BMI, body mass index.

**Table 2:** Features of the study population at time of celiac disease diagnosis in accordance with patatin-like phospholipase domain-containing protein 3 (PNPLA3) rs738409 genotype.

	CC (194)	CG (138)	GG (38)	*p*
Male/Female, *n* (%)	50 (25.8)/144 (74.2)	34 (24.7)/104 (75.3)	11 (29)/27 (71)	0.1
Age at diagnosis, years (mean ± SD)	31.3 ± 15.1	31.8 ± 16	29.8 ± 14.9	0.7
AST, U/mL (mean ± SD)	24.5 ± 27.7	24.6 ± 20.6	20.7 ± 7.6	0.6
ALT, U/mL (mean ± SD)	26.4 ± 23.6	25.1 ± 25.9	23.8 ± 23.7	0.7
Hypertransaminasemia, *n* (%)	22 (11.3)	13 (9.4)	4 (10.5)	0.2
Triglycerides, mg/dL (mean ± SD)	88.4 ± 39	90.3 ± 38	97.9 ± 29.6	0.3
Hypertriglyceridemia, *n* (%)	8 (4.1)	7 (5)	2 (5.2)	0.6
Total cholesterol, mg/dL (mean ± SD)	163.1 ± 36.6	165.6 ± 34.4	159.8 ± 31	0.6
Hypercholesterolemia, *n* (%)	22 (11.3)	19 (13.7)	5 (13.1)	0.7
HDL, mg/dL (mean ± SD)	55.4 ± 14	52.7 ± 12.1	55.4 ± 9.8	0.1
Fasting glucose, mg/dL (mean ± SD)	84 ± 20.2	85.2 ± 21	80.7 ± 11.8	0.4
Hyperglycemia, *n* (%)	5 (2.5)	2 (1.4)	1 (2.6)	0.3
Weight, kg (mean ± SD)	64.6 ± 13.1	64.5 ± 14	60 ± 15.2	0.1
BMI, kg/m^2^ (mean ± SD)	23.2 ± 3.5	23.4 ± 3.9	22.7 ± 3.2	0.5
Waist circumference, cm (mean ± SD)	84.8 ± 10.7	85.8 ± 11.5	84.4 ± 9.5	0.8
Hypertension, *n* (%)	18 (9.3)	15 (10.8)	5 (13.1)	0.2
Dyslipidemia, *n* (%)	25 (12.9)	17 (12.3)	4 (10.5)	0.3
Diabetes, *n* (%)	4 (2)	3 (2.1)	1 (2.6)	0.3
Metabolic syndrome, *n* (%)	14 (7.2)	11 (7.9)	3 (7.8)	0.9
Hepatic steatosis, *n* (%)	26 (13.9)	20 (14.4)	6 (15.7)	0.1

Statistical analysis between steatosis and no steatosis was performed by using Student’s *t*-test, Fisher’s exact test and ANOVA, when indicated. *n*, number; SD, standard deviation; AST, aspartate aminotransferase; ALT, alanine aminotransferase; HDL, high-density lipoprotein; BMI, body mass index.

**Table 3:** Features of the study population at follow-up in accordance with PNPLA3 rs738409 genotype.

	CC (194)	CG (138)	GG (38)	*p*
Male/Female, *n* (%)	50 (25.8)/144 (74.2)	34 (24.7)/104 (75.3)	11 (29)/27 (71)	0.1
Age at enrollment, years (mean ± SD)	36.7 ± 12.5	36.6 ± 12.9	36.1 ± 11.6	0.9
AST, U/mL (mean ± SD)	19.4 ± 7.3	19.3 ± 6.1	20.8 ± 6.9	0.4
ALT, U/mL (mean ± SD)	20.2 ± 10.8	20.5 ± 9.5	22.4 ± 10.4	0.4
Hypertransaminasemia, *n* (%)	10 (5.1)	6 (4.3)	4 (10.5)	0.1
Triglycerides, mg/dL (mean ± SD)	113.4 ± 53.7	113.5 ± 50.4	127.7 ± 59.7	0.2
Hypertriglyceridemia, *n* (%)	35 (18)	28 (20)	9 (23.6)	0.4
Total cholesterol, mg/dL (mean ± SD)	176.9 ± 33.3	178.8 ± 26.2	187.1 ± 38.2	0.1
Hypercholesterolemia, *n* (%)	47 (24.2)	33 (23.9)	12 (31.5)	0.3
HDL, mg/dL (mean ± SD)	55.7 ± 11.4	55.8 ± 15.6	47.7 ± 8.6	0.003
Fasting glucose, mg/dL (mean ± SD)	85.8 ± 14.5	87.7 ± 13.7	90.2 ± 10.4	0.1
Hyperglycemia, *n* (%)	18 (9.2)	8 (5.8)	4 (10.5)	0.2
Weight, kg (mean ± SD)	65.3 ± 12.3	67.8 ± 14.5	67.9 ± 11.7	0.9
BMI, kg/m^2^ (mean ± SD)	24.4 ± 3.4	24.6 ± 4.1	25 ± 2.9	0.6
Waist circumference, cm (mean ± SD)	85.9 ± 10.8	88.1 ± 12.6	87.1 ± 10	0.8
Hypertension, *n* (%)	44 (22.6)	51 (36.9)	18 (47.3)	0.001
Dyslipidemia, *n* (%)	51 (26.2)	49 (35.5)	18 (47.3)	0.009
Diabetes, *n* (%)	11 (5.6)	3 (2.1)	3 (7.8)	0.3
Metabolic syndrome, *n* (%)	44 (22.6)	50 (36.2)	18 (47.3)	0.001
Hepatic steatosis, *n* (%)	53 (27.3)	56 (40.5)	27 (71)	<0.001

Statistical analysis between steatosis and not steatosis was performed by using Student’s *t*-test, Fisher’s exact test and ANOVA, when indicated. *n*, number; SD, standard deviation; AST, aspartate aminotransferases; ALT, alanine aminotransferases; HDL, high-density lipoprotein; BMI, body mass index.

**Table 4:** Intergroup comparison in accordance with PNPLA3 rs738409 genotype.

	CC (194)		*p*	CG (138)		*p*	GG (38)		*p*
	At Diagnosis	At Follow-up		At Diagnosis	At Follow-up		At Diagnosis	At Follow-up	
AST, U/mL (mean ± SD)	24.5 ± 27.7	19.4 ± 7.3	0.01	24.6 ± 20.6	19.3 ± 6.1	0.004	20.7 ± 7.6	20.8 ± 6.9	0.9
ALT, U/mL (mean ± SD)	26.4 ± 23.6	20.2 ± 10.8	0.001	25.1 ± 25.9	20.5 ± 9.5	0.05	23.8 ± 23.7	22.4 ± 10.4	0.7
Hypertransaminasemia, *n* (%)	22 (11.3)	10 (5.1)	0.06	13 (9.4)	6 (4.3)	0.1	4 (10.5)	4 (10.5)	1
Triglycerides, mg/dL (mean ± SD)	88.4 ± 39	113.4 ± 53.7	<0.001	90.3 ± 38	113.5 ± 50.4	<0.001	97.9 ± 29.6	127.7 ± 59.7	0.007
Hypertriglyceridemia, *n* (%)	8 (4.1)	35 (18)	<0.001	7 (5)	28 (20)	<0.001	2 (5.2)	9 (23.6)	0.02
Total cholesterol, mg/dL (mean ± SD)	163.1 ± 36.6	176.9 ± 33.3	<0.001	165.6 ± 34.4	178.8 ± 26.2	<0.001	159.8 ± 31	187.1 ± 38.2	0.001
Hypercholesterolemia, *n* (%)	22 (11.3)	47 (24.2)	<0.001	19 (13.7)	33 (23.9)	0.03	5 (13.1)	12 (31.5)	0.05
HDL, mg/dL (mean ± SD)	55.4 ± 14	55.7 ± 11.4	0.8	52.7 ± 12.1	55.8 ± 15.6	0.06	55.4 ± 9.8	47.7 ± 8.6	0.001
Fasting glucose, mg/dL (mean ± SD)	84 ± 20.2	85.8 ± 14.5	0.3	85.2 ± 21	87.7 ± 13.7	0.2	80.7 ± 11.8	90.2 ± 10.4	<0.001
Hyperglycemia, *n* (%)	5 (2.5)	18 (9.2)	0.005	2 (1.4)	8 (5.8)	0.06	1 (2.6)	4 (10.5)	0.1
Weight, kg (mean ± SD)	64.6 ± 13.1	65.3 ± 12.3	0.6	64.5 ± 14	67.8 ± 14.5	0.06	60 ± 15.2	67.9 ± 11.7	0.01
BMI, kg/m^2^ (mean ± SD)	23.2 ± 3.5	24.4 ± 3.4	0.001	23.4 ± 3.9	24.6 ± 4.1	0.01	22.7 ± 3.2	25 ± 2.9	0.002
Waist circumference, cm (mean ± SD)	84.8 ± 10.7	85.9 ± 10.8	0.3	85.8 ± 11.5	88.1 ± 12.6	0.1	84.4 ± 9.5	87.1 ± 10	0.2
Hypertension, *n* (%)	18 (9.3)	44 (22.6)	<0.001	15 (10.8)	51 (36.9)	<0.001	5 (13.1)	18 (47.3)	0.001
Dyslipidemia, *n* (%)	25 (12.9)	51 (26.2)	<0.001	17 (12.3)	49 (35.5)	<0.001	4 (10.5)	18 (47.3)	<0.001
Diabetes, *n* (%)	4 (2)	11 (5.6)	0.07	3 (2.1)	3 (2.1)	1	1 (2.6)	3 (7.8)	0.3
Metabolic syndrome, *n* (%)	14 (7.2)	44 (22.6)	<0.001	11 (7.9)	50 (36.2)	<0.001	3 (7.8)	18 (47.3)	<0.001
Hepatic steatosis, *n* (%)	26 (13.9)	53 (27.3)	<0.001	20 (14.4)	56 (40.5)	<0.001	6 (15.7)	27 (71)	<0.001

Statistical analysis between steatosis and no steatosis was performed by using Student’s *t*-test, Fisher’s exact test and ANOVA, when indicated. *n*, number; SD, standard deviation; AST, aspartate aminotransferase; ALT, alanine aminotransferase; HDL, high-density lipoprotein; BMI, body mass index.

**Table 5:** Comparison of macronutrient intake in celiac patients with different PNPLA3 genotypes, with or without steatosis.

	CC (194)	CG (138)	GG (38)	*p*	Steatosis (136)	No Steatosis (234)	*p*
Energy, kcal (mean ± SD)	2210 ± 366	2234 ± 359	2267 ± 382	0.6	2261 ± 363	2239 ± 359	0.3
Carbohydrates, g (mean ± SD)	243.4 ± 51	245.2 ± 50	248.1 ± 53	0.8	247.2 ± 49	244.8 ± 52	0.6
Fat, g (mean ± SD)	36.7 ± 5.5	36.6 ± 5.9	36.8 ± 5.6	0.9	36.8 ± 5.7	36.7 ± 5.5	0.8
Protein, g (mean ± SD)	75.2 ± 15.6	74.8 ± 16.4	72.9 ± 15.9	0.7	75.1 ± 16.2	75.2 ± 16	0.9
Fiber, g (mean ± SD)	24.4 ± 5.6	25.1 ± 5.9	24.8 ± 5.7	0.5	24.7 ± 5.8	24.8 ± 5.7	0.8

Fifty-six (41%) patients with the CG genotype and 27 (71%) with the GG genotype showed HS on ultrasound, compared to 53 (27%) patients with the CC genotype (*p* < 0.001). When stratified by BMI, however, the prevalence of HS in subjects with different PNPLA3 genotypes showed no statistically significant difference between groups.

**Table 6:** Baseline factors associated with (**A**) hepatic steatosis and (**B**) metabolic syndrome.

	**Univariate Analysis**			**Binary Logistic Regression**		
**Variable**	OR	95% CI	*p*	OR	95% CI	*p*
**Gender**	1.61	1–2.6	**0.04**	1.1	0.8–1.5	0.4
**G allele (heterozygous genotype)**	2.37	1.5–3.6	**<0.001**	1.9	1.4–2.7	**<0.001**
**GG alleles (homozygous genotype)**	6.53	3–14	**<0.001**	6.9	4.2–9.5	**<0.001**
**Hypertransaminasemia at CD diagnosis**	1.95	1–3.8	**0.04**	1.8	1.1–3.2	0.5
**Hypertriglyceridemia at CD diagnosis**	2	0.7–5.3	0.1			
**Hypercholesterolemia at CD diagnosis**	2.28	1.2–4.2	**0.008**	2	1.2–4.3	0.4
**Hyperglycemia at CD diagnosis**	2.9	0.6–12	0.1			
**BMI at CD diagnosis**	2.2	1.4–4.7	**<0.001**	1.02	0.4–2.1	0.7
**Waist circumference at CD diagnosis**	1.16	0.9–1.4	0.1			
	**Univariate Analysis**			**Binary Logistic Regression**		
**Variables**	OR	95% CI	*p*	OR	95% CI	*p*
**Gender**	2.79	1.7–4.5	**<0.001**	1.2	0.7–2.21	0.7
**G allele (heterozygous genotype)**	2.14	1.3–3.3	**0.001**	1.3	0.8–2.3	0.1
**GG alleles (homozygous genotype)**	3	3.4–6.3	**0.002**	1.9	1.2–4.7	0.1
**Hypertransaminasemia at CD diagnosis**	2.73	1.3–5.3	**0.003**	1.2	0.6–2.21	0.6
**Hypertriglyceridemia at CD diagnosis**	2.5	1–6.5	**0.037**	1.1	0.4–2.5	0.8
**Hypercholesterolemia at CD diagnosis**	1.5	0.8–2.9	0.1			
**Hyperglycemia at CD diagnosis**	3.9	0.9–16	**0.045**	1.17	0.33–4	0.8
**BMI at CD diagnosis**	2.8	1.6–5.2	**<0.001**	3.8	1.6–5.2	**<0.001**
**Waist circumference at CD diagnosis**	3.2	1.8–6.4	**0.005**	2.8	1.9–5.4	**0.03**

CD, celiac disease; BMI, body mass index; OR; odds ratio; CI, confidence interval. Significant *p* values have been highlighted in bold. Regression model used a backward stepwise selection (Wald) method.
